# Supplementary material for: Direct conversion of methane to aromatics and hydrogen via a heterogeneous trimetallic synergistic catalyst
Source: Nat Commun. 2024 Apr 16;15:3280. doi: 10.1038/s41467-024-47595-9 (PMC11021476; doi:10.1038/s41467-024-47595-9)
Supplement: Supplementary file 1 — Supplementary Information [file 41467_2024_47595_MOESM1_ESM.pdf]

Supporting Information

Direct conversion of methane to aromatics and hydrogen via a heterogeneous trimetallic synergistic catalyst

Pengxi Zhu[1,2], Wenjuan Bian[1], Bin Liu[3], Hao Deng[3], Lucun Wang[1], Xiaozhou Huang[2], Stephanie L. Spence[4], Feng Lin[4], Chuancheng Duan[3], Dong Ding[1,*], Pei Dong[2,*], Hanping Ding[1,5*]

Affiliations:

[1] Energy and Environment Science & Technology, Idaho National Laboratory, Idaho Falls, ID 83415 USA.

[2] Department of Mechanical Engineering, George Mason University, Fairfax, VA 22030 USA.

[3] Department of Chemical Engineering, Kansas State University, Manhattan, KS 66506 USA.

[4] Department of Chemistry, Virginia Tech, Blacksburg, VA 24061 USA.

[5] School of Aerospace and Mechanical Engineering, University of Oklahoma, Norman, OK 73019 USA.

Correspondence authors' email: dong.ding@inl.gov; pdong3@gmu.edu; hding@ou.edu

Equations S1 for calculation of methane conversion, product relative and absolute selectivity, and yield.

$$\text{Methane conversion} = \frac{mol\ of\ methane\ inlet - mol\ of\ methane\ outlet}{mol\ of\ methane\ inlet} * 100\% \quad (1)$$

$$\text{Product relative selectivity} = \frac{N_{carbon} * mol\ of\ each\ component\ of\ formed\ product}{mol\ of\ formed\ product} * 100\% \quad (2)$$

$$\text{Product absolute selectivity} = \frac{N_{carbon} * mol\ of\ each\ component\ of\ formed\ product}{mol\ of\ conversed\ methane} * 100\% \quad (3)$$

$$\text{Product yield} = methane\ conversion\ * \frac{Product\ absolute\ selectivity}{100\%} \quad (4)$$

N_{carbon}: The carbon number of product being formed. e.g., N_{carbon} is 6 for C_6H_6.

Equations S2 for calculation of coke in the spent catalyst.

$$Cokeamount\ \left(\frac{mg}{g}.cat\right) = \frac{M1 - M2}{M2} \times 1000$$

Where M1 denotes the weight percent of the coked catalyst before coke burn-off, and M2 denotes the weight percent of the coked catalyst after coke burn-off.

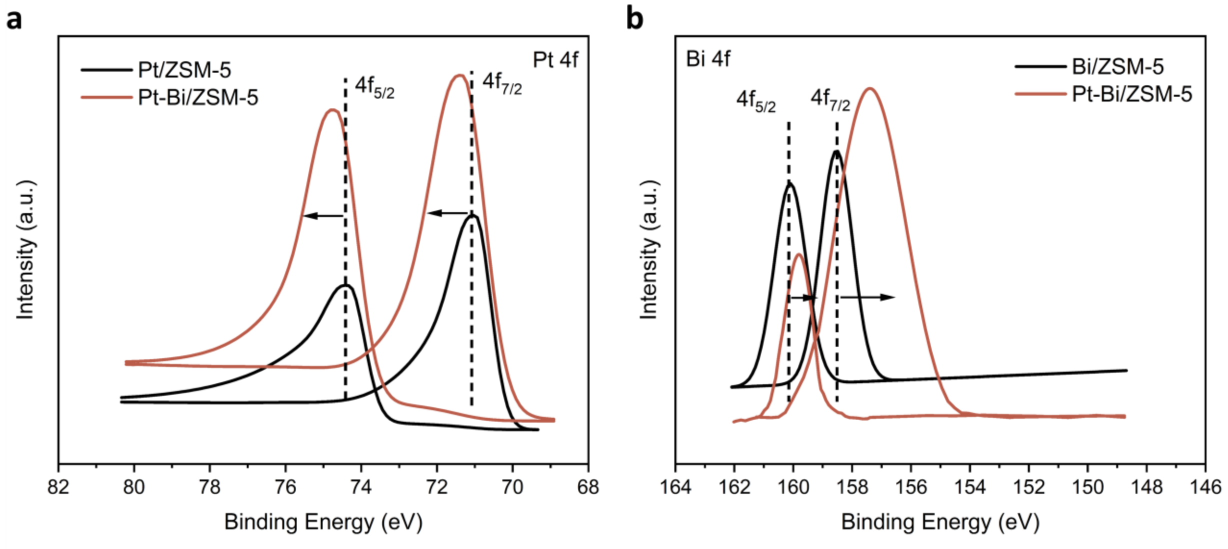

Figure S1. XPS fitting spectra of (a) Pt 4f and (b) Bi 4f from the as-prepared Pt/ZSM-5, Bi/ZSM5 and Pt-Bi/ZSM-5 samples.

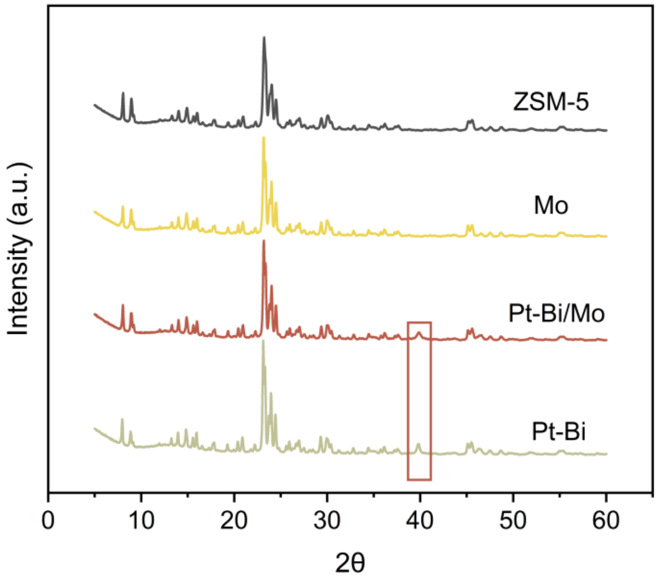

Figure S2. XRD pattern for fresh different supported and unsupported ZSM-5.

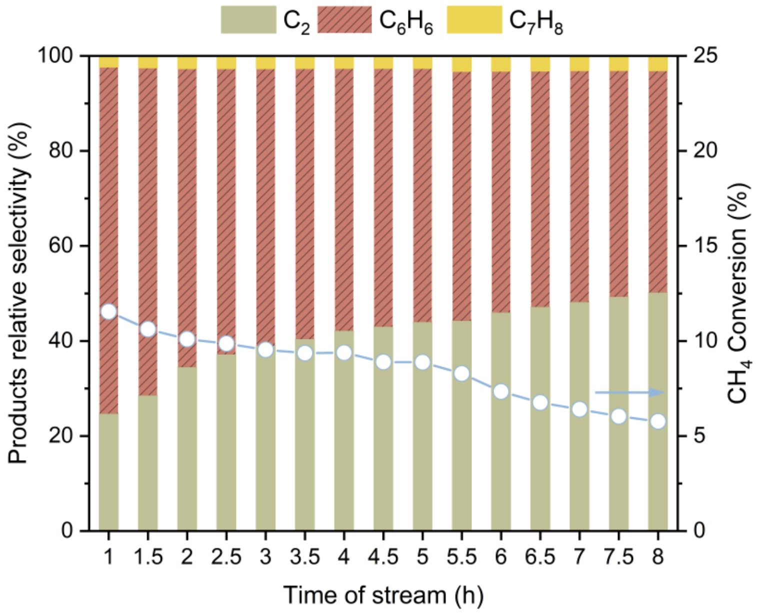

Figure S3. The catalytic performance of 0% ratio of Bismuth in Pt-Bi/Mo catalyst.

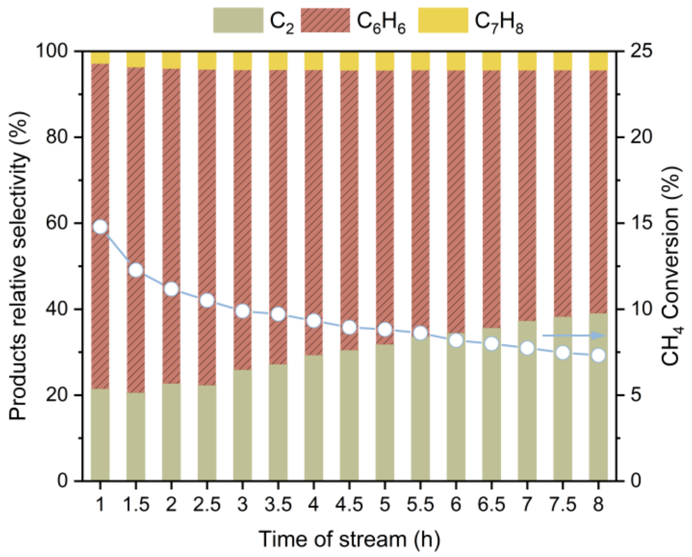

Figure S4. The catalytic performance of 0.3% ratio of Bismuth in Pt-Bi/Mo catalyst.

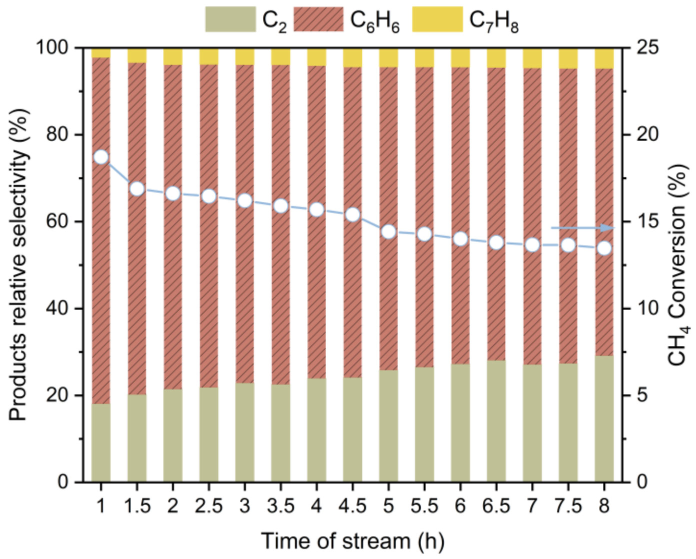

Figure S5. The catalytic performance of 0.6% ratio of Bismuth in Pt-Bi/Mo catalyst.

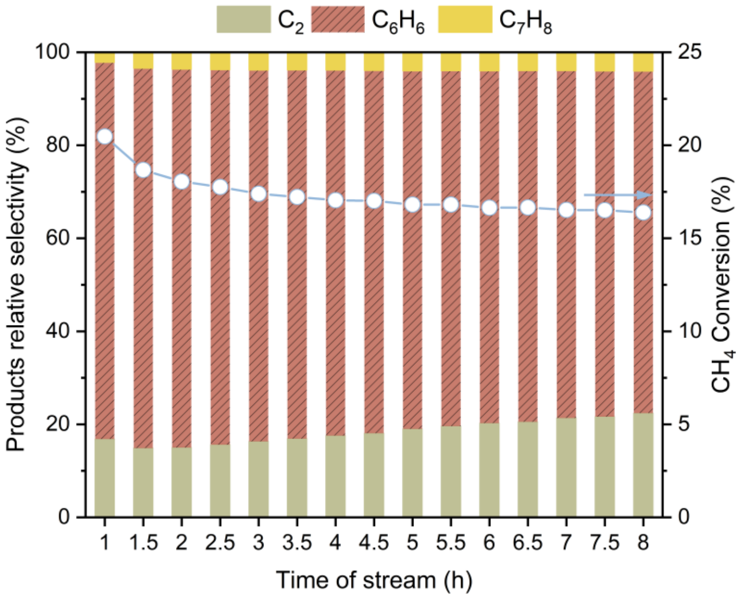

Figure S6. The catalytic performance of 0.8% ratio of Bismuth in Pt-Bi/Mo catalyst.

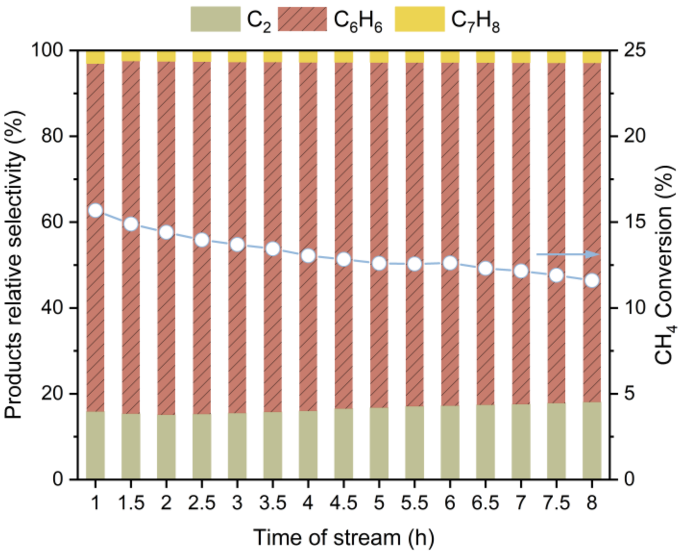

Figure S7. The catalytic performance of 1% ratio of Bismuth in Pt-Bi/Mo catalyst.

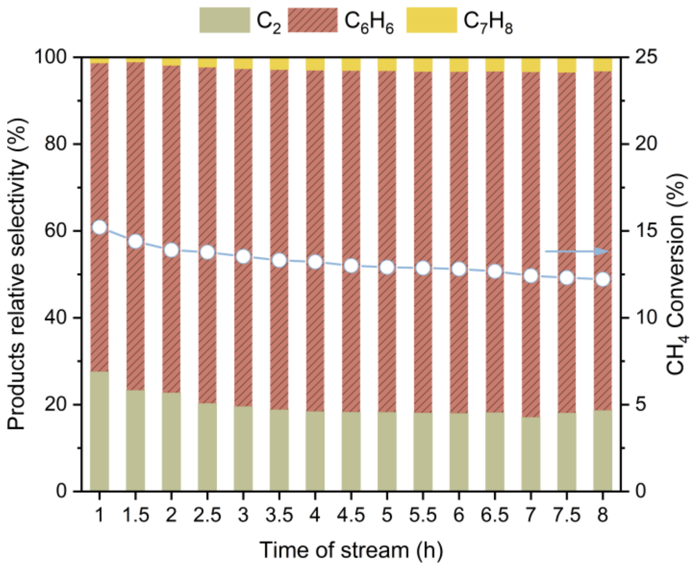

Figure S8. The catalytic performance of 1.5% ratio of Bismuth in Pt-Bi/Mo catalyst.

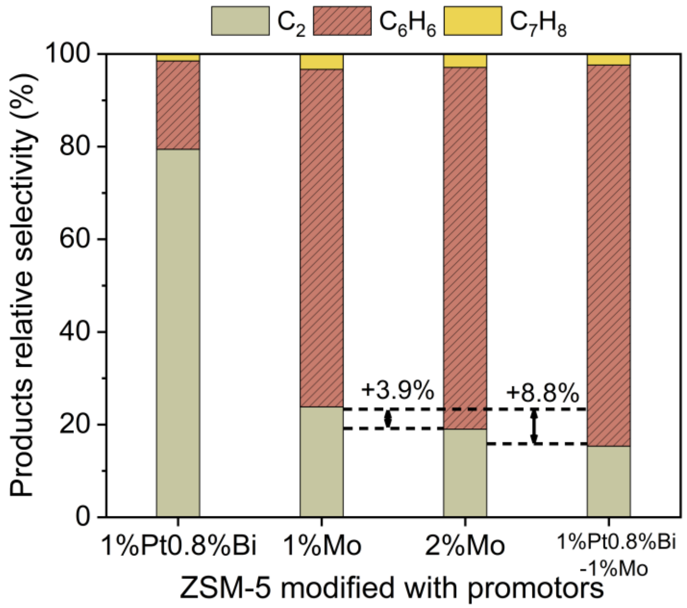

Figure S9. The product relative selectivity for ZSM-5 modified with promoters, tested at 710 °C, 0.95 atm CH_4, and 1272 mL·gcat^{-1}·h^{-1}.

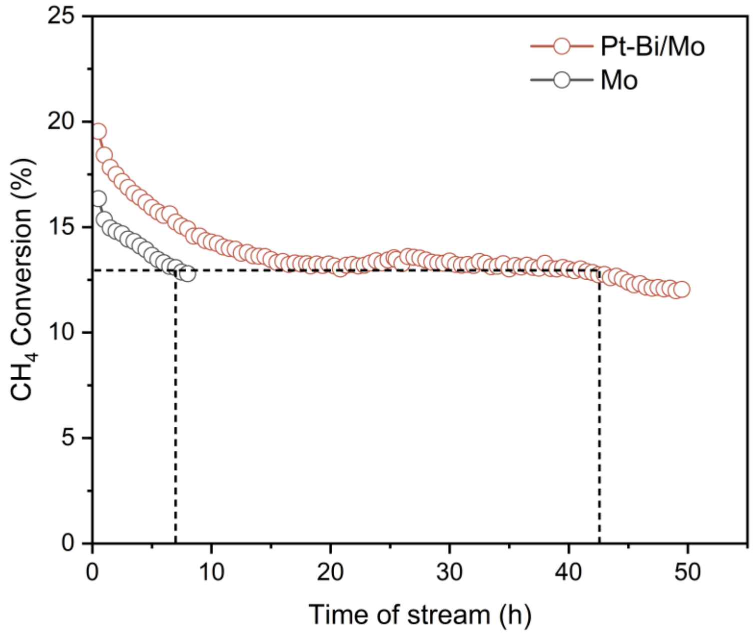

Figure S10. The long-term testing comparison between Pt-Bi/Mo catalyst and Mo catalyst.

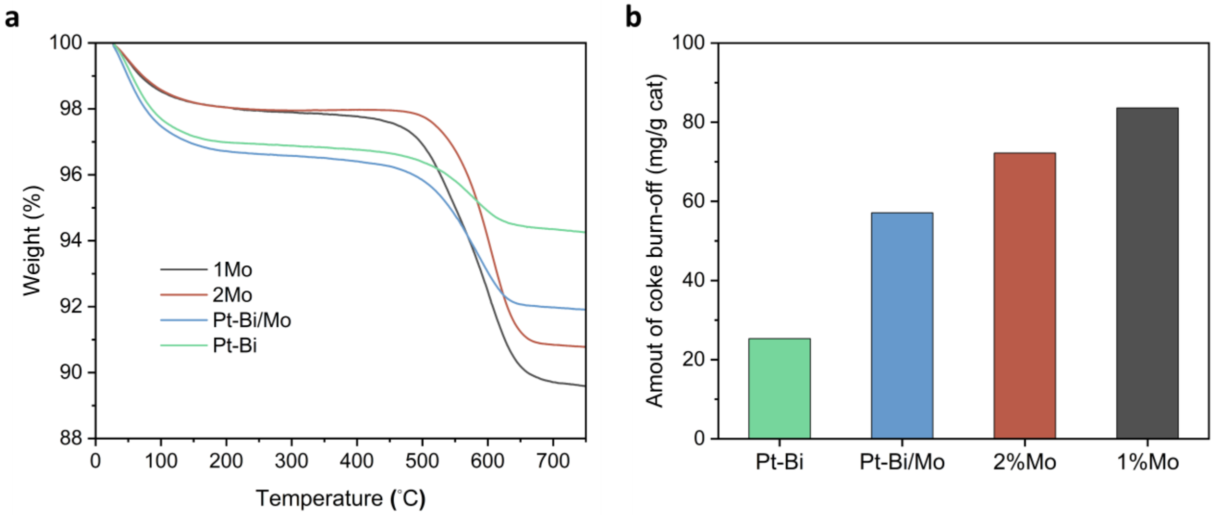

Figure S11. a, TGA profiles of different spent catalysts after 8h TOS at 710 °C; b, Amount of calculated coke burn-off in spent catalyst.

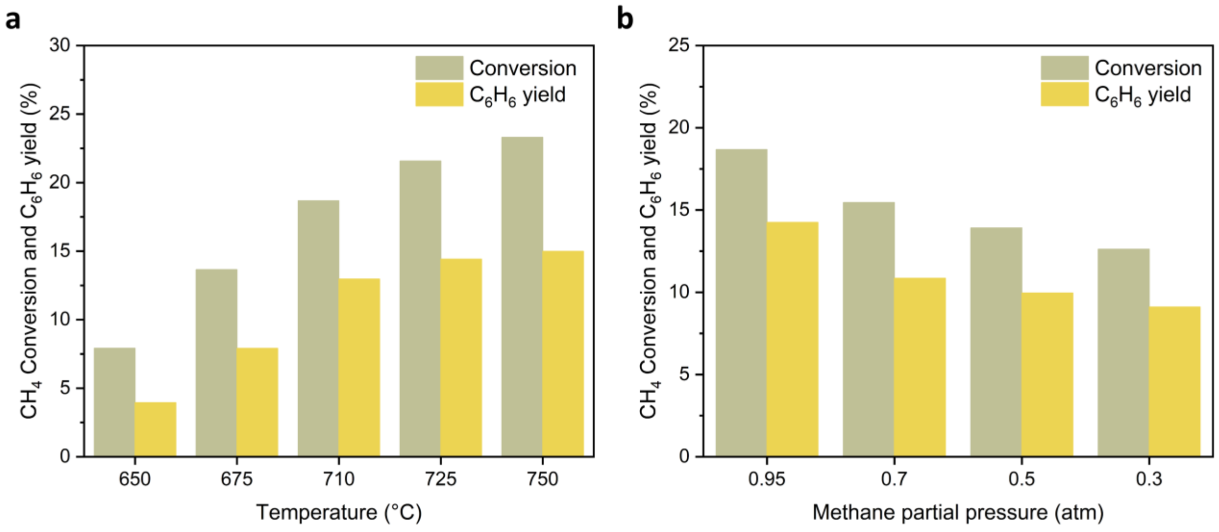

Figure S12. The effects of operating conditions on catalytic performance. a, temperature; b, methane partial pressure.

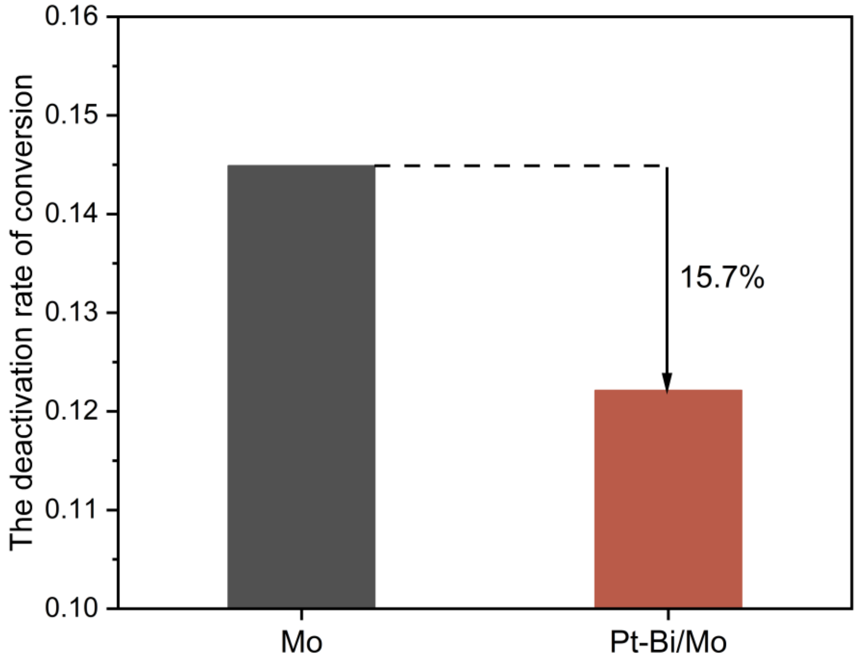

Figure S13. The deactivation rate of conversion comparison between Pt-Bi/Mo catalyst and Mo catalyst.

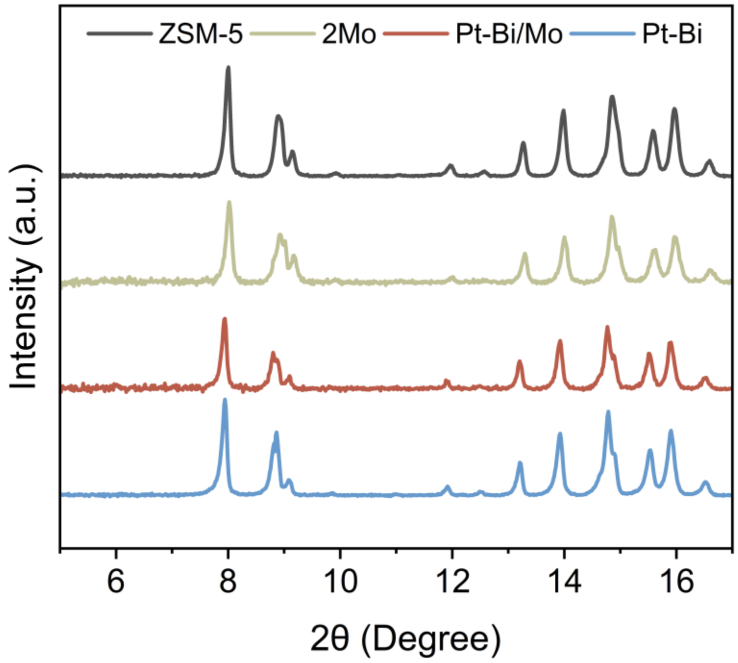

Figure S14. Low-angle X-ray diffraction (XRD) pattern for different spent catalysts calcined in CH_4 for 120 min.

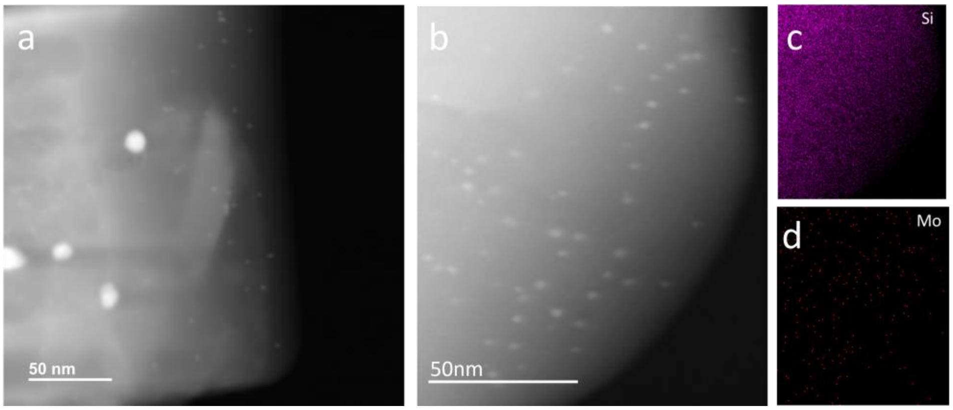

Figure S15. HAADF -STEM images of (a) Pt-Bi/Mo/ZSM-5 and (b) Mo/ZSM-5 after reaction for 120 minutes and relative EDS images of Mo/ZSM-5.

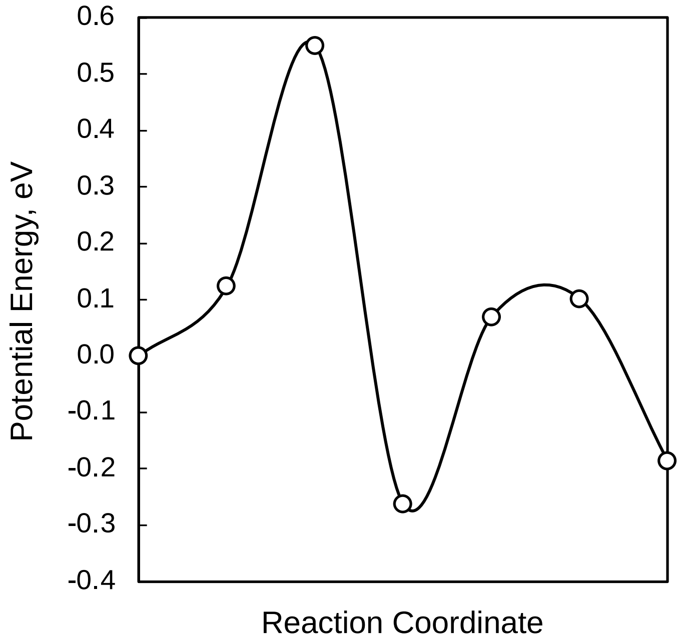

Figure S16. Potential energy surface for CH_4 activation at the Mo_2C-PtBi interface.

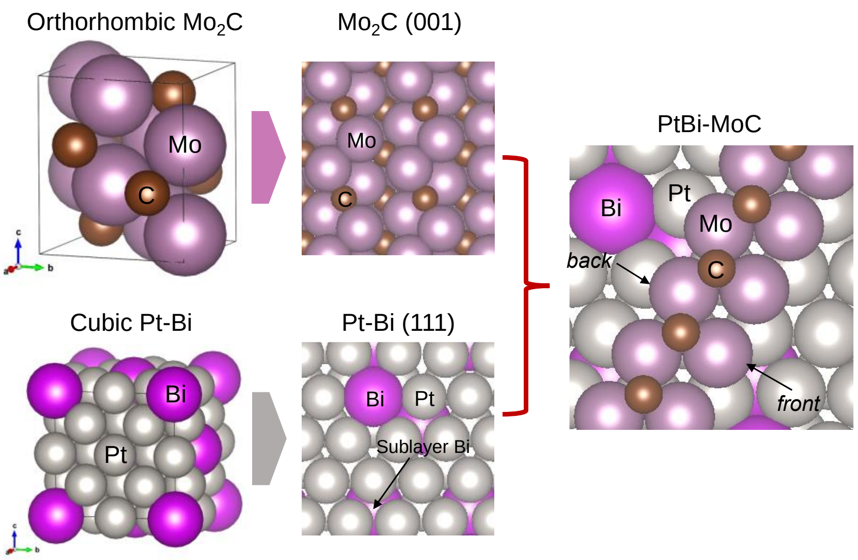

Figure S17. Construction of catalyst models. Relaxed bulk Mo_2C and Pt-Bi crystals are cleaved to expose their (001) and (111) facets, respectively. The PtBi-MoC composite was built by placing a periodic 1D Mo_2C nanowire on Pt-Bi(111) to represent the Mo_2C and Pt-Bi co-catalyst. Color scheme: Mo (purple), C (brown), Bi (magenta), and Pt (grey).

Supplementary Table 1. Comparison of MDA activities for photocatalysts and thermocatalysts include various supported metals on zeolite and iron on silicon dioxide.

Catalysts	Temperature (°C)	WHSV ($mL·g_{cat}^{-1}·h^{-1}$)	CH_4 conversion (%)	Benzene selectivity(%)	Reference
Pt-Bi/Mo	710	1272	18.7	69.4	
V	750	800[a]	3.2	32.6	[1]
Zn	700	3000	0.9	88.9	[2]
W	800	_[b]	8.7	26.4	[3]
Re	700	4500	10.5	49.2	[4]
Fe	700	4500	6.6	27.4	[4]
Ni	700	3000	4.5	35.6	[5]
Mn	750	_[b]	5.4	50.4	[6]
Mo	730	1500	16.7	60.4	[7]
Mo	700	2000	12.8	71.1	[8]
Fe-Mo	700	1550	9.5	46.3	[9]
Pt-Mo	750	2700	7.2	71.6	[10]
Co-Mo	700	_[b]	14.9	46.9	[11]
Ni-Mo	700	_[b]	16.2	39.5	[11]
Ga-Mo	700	840	11.9	69	[12]
Rh-Mo	750	_[b]	25	43.2	[13]
Pt-Sn/Mo	700	1620	5.6	77	[14]
p-type GaN	5	_[b]	0.07	0.07	[15]
n-type GaN	5	_[b]	0.98	0.95	[15]
Fe©SiO_2	1020	4840	32.0	6.9	[16]
Fe/SiO_2	950	4840	14.0	3.4	[16]
Fe/SiC	950	4840	11.0	2.0	[16]

a: GHSV/h^{-1}; b: Not reported

Supplementary Table 2. Surface area and pore volume of the Spent Pt-Bi/Mo and Mo-based catalysts.

Sample	S_{BET} (m^2 g^{-1})[a]	S_{micro} (m^2/g)[b]	S_{exter} (m^2/g)[b]	V_{micro} (cc g^{-1})[b]	V_{total} (cc g^{-1})[c]
**ZSM-5**	509	311	198	0.163	0.366
**1%Mo**	356	212	134	0.114	0.227
**2%Mo**	329	202	152	0.106	0.22
**Pt-Bi**	367	217	151	0.119	0.256
**Pt-Bi/Mo**	354	198	134	0.102	0.209

a Calculated using BET method

b Calculated using t-plot method

c Calculated using DFT method

Supplementary Table 3. The fitting report about Napierian logarithm of reaction rate constant (Arrhenius curve) as a function of reaction temperature reciprocal.

Plot	2%Mo	1%Mo	Pt-Bi/Mo	Pt-Bi
**Intercept**	-0.36472 ± 0.41158	-0.76764 ± 0.68119	-5.63113 ± 0.78877	3.19808 ± 1.26647
**Slope**	-8056.6874 ± 374.27585	-7951.22569 ± 619.45024	-3262.17617 ± 717.27583	-12471.18448 ± 1151.67923
**Residual Sum of Squares**	0.00128	0.0035	0.00469	0.0121
**Pearson's r**	-0.99785	-0.99399	-0.9549	-0.99158
**R-Square (COD)**	0.9957	0.98801	0.91183	0.98323

The binding energies and site preferences were obtained from DFT calculations (see the Computational Method section) to distinguish the catalytic roles of Pt-Bi alloy and the Mo_2C in the system. The binding energies (BE) were calculated based on Equation (S3).

$$BE_{adsorbate} = E_{adsorbate} - E_{clean\ surface} - E_{molecule(g)} \qquad S3$$

where $E_{adsorbate}$ and $E_{clean\ surface}$ represent the total energy of surface adsorbate, clean surface, respectively. $E_{molecule(g)}$ corresponds to the total energy of molecule that is stable in the gas phase. In this work, the total energies of H_2 and CH_4 were used for the H element and CH_3/CH_2.

Table S4. Binding energies (in eV) and site preference on Pt-Bi(111) and Mo_2C(001).

Species	Pt-Bi(111)		Mo_2C(001)	
	BE (eV)	Site preference	BE (eV)	Site preference
CH_4	-0.02	Bi top	-0.02	Mo top
CH_3	0.32	Pt top	-0.12	Mo-Mo bridge
CH_2	0.91	Pt-Pt bridge	0.26	Mo-Mo bridge
C_2H_4	-1.33	Pt-Pt bridge	-1.97	Mo-Mo bridge
H	-0.52	3-fold fcc Pt	-0.92	Mo-Mo bridge

Supplementary References:

1. Weckhuysen, B.M., et al., *Conversion of Methane to Benzene over Transition Metal Ion ZSM-5 Zeolites: I. Catalytic Characterization.* Journal of Catalysis, 1998. **175**(2): p. 338-346.
2. Abdelsayed, V., M.W. Smith, and D. Shekhawat, *Investigation of the stability of Zn-based HZSM-5 catalysts for methane dehydroaromatization.* Applied Catalysis A: General, 2015. **505**: p. 365-374.
3. Çağlayan, M., et al., *Understanding W/H-ZSM-5 catalysts for the dehydroaromatization of methane.* Catalysis Science & Technology, 2023. **13**(9): p. 2748-2762.
4. Liu, Y., et al., *Transition-Metal Catalysts for Methane Dehydroaromatization (Mo, Re, Fe): Activity, Stability, Active Sites, and Carbon Deposits.* ACS Catalysis, 2023. **13**(1): p. 1-10.
5. Xu, Y., et al., *CH4 conversion over Ni/HZSM-5 catalyst in the absence of oxygen: decomposition or dehydroaromatization?* Chemical Communications, 2020. **56**(32): p. 4396-4399.
6. Lim, T.H. and D.H. Kim, *Characteristics of Mn/H-ZSM-5 catalysts for methane dehydroaromatization.* Applied Catalysis A: General, 2019. **577**: p. 10-19.
7. Su, L., et al., *Creating Mesopores in ZSM-5 Zeolite by Alkali Treatment: A New Way to Enhance the Catalytic Performance of Methane Dehydroaromatization on Mo/HZSM-5 Catalysts.* Catalysis Letters, 2003. **91**(3): p. 155-167.
8. Kosinov, N., et al., *Methane Dehydroaromatization by Mo/HZSM-5: Mono- or Bifunctional Catalysis?* ACS Catalysis, 2017. **7**(1): p. 520-529.
9. Hossain, M.S., et al., *Elucidating the role of Fe-Mo interactions in the metal oxide precursors for Fe promoted Mo/ZSM-5 catalysts in non-oxidative methane dehydroaromatization.* Chemical Engineering Journal, 2023. **475**: p. 146096.
10. Kojima, R., et al., *Promotion effects of Pt and Rh on catalytic performances of Mo/HZSM-5 and Mo/HMCM-22 in selective methane-to-benzene reaction.* Catalysis Letters, 2006. **110**(1): p. 15-21.
11. Sridhar, A., et al., *Bimetallic Mo-Co/ZSM-5 and Mo-Ni/ZSM-5 catalysts for methane dehydroaromatization: A study of the effect of pretreatment and metal loadings on the catalytic behavior.* Applied Catalysis A: General, 2020. **589**: p. 117247.
12. Liu, B.S., et al., *XPS, XAES, and TG/DTA characterization of deposited carbon in methane dehydroaromatization over Ga–Mo/ZSM-5 catalyst.* Applied Surface Science, 2007. **253**(11): p. 5092-5100.
13. Ramasubramanian, V., et al., *Effect of Addition of K, Rh and Fe Over Mo/HZSM-5 on Methane Dehydroaromatization Under Non-oxidative Conditions.* Catalysis Letters, 2019. **149**(4): p. 950-964.
14. Tshabalala, T.E., et al., *Dehydroaromatization of methane over Sn–Pt modified Mo/H-ZSM-5 zeolite catalysts: Effect of preparation method.* Applied Catalysis A: General, 2015. **503**: p. 218-226.
15. Li, L., et al., *Photoinduced Conversion of Methane into Benzene over GaN Nanowires.* Journal of the American Chemical Society, 2014. **136**(22): p. 7793-7796.
16. Guo, X., et al., *Direct, Nonoxidative Conversion of Methane to Ethylene, Aromatics, and Hydrogen.* Science, 2014. **344**(6184): p. 616-619.
